# Platelet PI3Kγ Contributes to Carotid Intima-Media Thickening under Severely Reduced Flow Conditions

**DOI:** 10.1371/journal.pone.0129265

**Published:** 2015-06-08

**Authors:** Cuiping Wang, Rong Jin, Anil Nanda, Jinchuan Yan, Guohong Li

**Affiliations:** 1 Department of Cardiolog, Affiliated Hospital of Jiangsu University, Zhenjiang, Jiangsu Province, 212001, P. R. China; 2 Department of Neurosurgery, LSU Health Science Center, Shreveport, LA, 71103, United States of America; Baker IDI Heart and Diabetes Institute, AUSTRALIA

## Abstract

Studies have begun to focus on the emerging function of platelets as immune and inflammatory cells that initiate and accelerate vascular inflammation. Phosphoinositide 3-kinase gamma (PI3Kγ) is critically involved in a number of inflammatory and autoimmune diseases. This study aims to investigate the contribution of platelet PI3Kγ to vascular remodeling under flow severely reduced conditions. Mouse partial left carotid artery ligation with adoptive transfer of activated, washed wild-type or PI3Kγ^-/-^ platelets was used as the model. Intima-media area, leukocyte recruitment, and proinflammatory mediator expression were assessed. In vitro PI3Kγ^-/-^ platelets were used to verify the effect of PI3Kγ on platelet activation, interaction with leukocytes, and endothelial cells. Mice injected with activated platelets showed a significant increase in intima-media thickening, recruitment of neutrophils (at 3 d) and macrophages (at 21 d), and intercellular adhesion molecule-1, vascular cell adhesion molecule-1, tumor necrosis factor alpha, and interleukin-6 expression (at 3 d) in the flow-reduced area. These effects were abrogated by platelet PI3Kγ deficiency. Circulating platelet-leukocyte aggregates were reduced in PI3Kγ^-/-^ mice after partial ligation. In vivo data confirmed that PI3Kγ mediated Adenine di-Phosphate -induced platelet activation through the Akt and p38 MAP kinase signaling pathways. Moreover, platelet PI3Kγ deficiency reduced platelet-leukocyte aggregation and platelet-endothelial cell (EC) interaction. These findings indicate that platelet PI3Kγ contributes to platelet-mediated vascular inflammation and carotid intima-media thickening after flow severely reduced. Platelet PI3Kγ may be a new target in the treatment of vascular diseases.

## Introduction

The function of platelets in atherosclerosis is well established. Platelets are well known to participate in the final step of atherosclerosis, that is, plaque rupture followed by thrombotic narrowing or occlusion of a vessel. However, platelet involvement in the earliest processes of vascular inflammation is increasingly being recognized [1]. Activated platelets are found to be present in the circulating blood of patients with unstable atherosclerosis, stable coronary disease, and hypercholesterolemia [2–4]. Circulating activated platelets can affect endothelial inflammation and leukocyte-endothelial interaction, as well as exacerbate atherosclerosis. Research found that repeated injections of activated platelets into mouse increased the size of atherosclerotic lesions [5].

Vascular remodeling of the carotid artery, clinically defined as carotid intima-media thickening (CIMT), is a strong predictor for future vascular events in the general population [6]. Lorenz et al. performed a meta-analysis showing that for an absolute carotid IMT difference of 0.1 mm, the future risk of myocardial infarction increases by 10% to 15%, and the stroke risk increases by 13% to 18% [7]. Vascular remolding preferentially occurs at particular areas of disturbed flow characterized by low and oscillatory wall shear stress (WSS) in branched or curved arteries [8]. Research on the function of activated platelets in vascular remodeling under flow severely reduced conditions remains lacking.

Partial carotid ligation was found to be a model of acutely induced disturbed flow, leading to rapid endothelial dysfunction and vascular remodeling [9]. This model involves a significant reduction in flow in the ligated left common carotid artery (LCA) with maintenance of an intact endothelium and no thrombosis, in contrast to the complete ligation of the common carotid artery, which results in no flow through LCA and may be associated with endothelial denudation and thrombosis. Thus, this model is appropriate for studying the function of platelets in vascular remodeling.

Phosphoinositide 3-kinases (PI3Ks), a family of enzymes characterized by protein and lipid kinase activity, are divided into three classes on the basis of their primary structure, mode of regulation, and substrate specificity as classes I, II, and III. PI3K class I family members have a p110 catalytic subunit, and these p110 subunits associate with regulatory subunits. Class IA PI3Ks have three p110 catalytic subunits: p110α, p110β, and p110δ. The only member of the PI3K class IB family is PI3Kγ. Increasing evidence reveals that PI3Kγ is critically involved in a number of inflammatory and autoimmune diseases [10–12].

Platelets express three of the four cell surface receptor-activated “class I” PI3K isoforms: PI3Kα, PI3Kβ, and PI3Kγ [13], but not PI3Kδ. PI3Kγ is activated by Gβγ subunits of heterotrimeric G proteins in vitro and has thus been suggested to relay signals from G protein-coupled receptors (GPCRs) [14, 15]. Many agonists involved in platelet functions act via the GPCRS. Thus, PI3Kγ might be an important contributor to platelet function.

In this study, we injected activated, washed platelets to investigate their function under severely reduced-flow conditions using the mouse partial carotid ligation model. Further, we explored the crucial function of platelet PI3Kγ in vascular remolding and the potential mechanism involved.

## Materials and Methods

### Animals

All experimental procedures were carried out in accordance with the NIH Guide for the Care and Use of Laboratory Animals and approved by the Animal Care and Use Committee of the Louisiana State University Health Science Center-Shreveport (IACUC approval number: 0819). C57BL/6J mice (wild type [WT]) were purchased from Jackson Laboratories (Bar Harbor, Maine). PI3K-p110γ knockout (PI3Kγ^-/-^) mice on the C57BL/6J background were made by J.M.P. [16], transferred to the LSU Health Sciences Center-Shreveport (Louisiana), and housed in a specific pathogen-free environment. Male mice (8 to 10 weeks old) were used in this study.

### Partial left carotid artery ligation (PLCA)

PLCA surgery was performed as previously reported [9, 17, 18]. In brief, mice were anesthetized with intraperitoneal administration of ketamine (80 mg/kg body wt; Abbott Laboratories) and xylazine (5 mg/kg body wt; Rompun, Bayer Corp). Then, a ventral midline incision of 4 mm to 5 mm was made in the neck. With the use of blunt dissection, muscle layers were separated with curved forceps to expose the LCA. Under a light microscope, three (left external carotid, internal carotid, and occipital artery) out of four branches of the LCA were ligated using a 7–0 silk suture. The superior thyroid artery was left intact to be the sole source of blood circulation. The incision was then closed and cleaned with betadine. The animals were kept on a heating pad until they gained consciousness.

### Donor platelets preparation and injection

Platelets were prepared as described previously [19]. Approximately 0.9 ml of blood was harvested via the carotid artery into a polypropylene tube containing 0.1 ml of acid-citrate-dextrose buffer (Sigma). Platelet-rich plasma was obtained by two sequential centrifugations (120 g for 8 min and 120 g for 3 min). Platelets were pelleted at 550 g for 10 min and resuspended in PBS (pH 7.4). Platelet count was adjusted to 2 × 10^8^/ml. Washed platelets were activated with 20 μM ADP (Sigma) for 15 min, pelleted at 550 g for 10 min, and resuspended in PBS. Activated wild-type (WT) platelets or PI3Kγ^-/-^ platelets (3×10^7^/20 g body weight) were administered every 5 d to C57BL/6 acceptor mice via tail vein injections [5]. The first injection was 2 d before surgery.

### Tissue harvesting

Mice were euthanized at 3 or 21 d after surgery, and vessels were perfused with PBS under physiological pressure followed by perfusion with 4% paraformaldehyde (PFA) and post-fixed with 4% PFA overnight. For immunofluorescence staining, the arteries were excised and embedded in OCT compound (Tissue-Tek), prepared as frozen slides (10 μm thick sections), and stored at −80°C pending analysis. For neointimal assessment, the arterial segments were embedded in paraffin and mounted on slides (5 μm thick sections).

### Morphometric analysis

**Fig 1:**
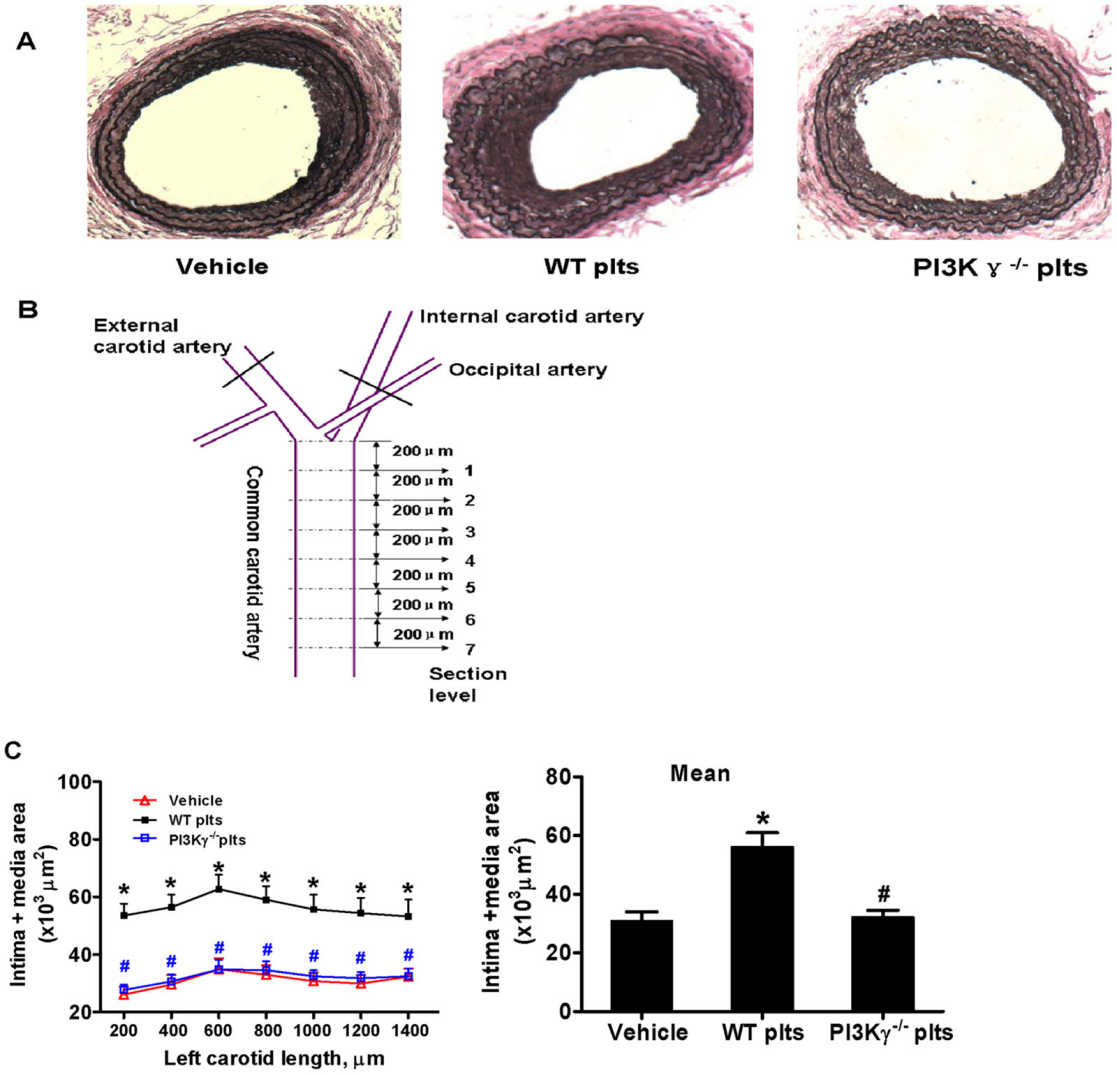
Platelet PI3Kγ contributes to activated platelets induced intima-media thickening after partial ligation. (A) Representative Verhoeff’s elastic stained cross sections (level 3) of left common carotid arteries from WT mice treated with PBS (Vehicle), WT platelets (WT plts), or PI3Kγ^-/-^ platelets (PI3Kγ^-/-^ plts) (n = 5 per group) at 21 d after partial ligation. (B) Schematic diagram of partial ligation and tissue microtomy. Intima+ media areas (C) were measured at seven section levels (200μm intervals), and their mean values were determined. Data are expressed as mean ± SEM. * *P*<0.05 versus vehicle, # *P*<0.05 versus WT platelet-infused mouse. Light microscope magnification is 10×.

Serial cross-sections (5 μm thick) were cut beginning 200 μm proximal to the carotid bifurcation. Histomorphometric analysis was performed at seven cross-section levels (with 200 μm intervals) (Fig 1B). For each level, three cross-sections were stained with Verhoeff’s elastic staining, and morphometric analysis was performed by an individual blinded to the experimental design. We measured the lumen area, intima area [the area within the internal elastic lamina (IEL) minus the lumen area], and medial area [defined as the area within the external elastic lamina (EEL) minus the area within the IEL] at each level as previously described [20, 21]. The mean value of intima and media area was calculated over seven cross-section levels.

### Immunofluorescence staining

Immunofluorescence staining was performed on frozen sections using the following antibodies: intercellular adhesion molecule-1 (ICAM-1) (1:200; Abcam) and vascular cell adhesion molecule-1 (VCAM-1) (1:200; Abcam). Mac-2 (1:1000; M3/38, Accurate) was used to detect monocyte/macrophages, whereas anti-PMN mAb (1:2500; Accurate) was used to detect neutrophils. Isotype-matched antibodies served as negative controls. Sections were incubated with the indicated antibodies overnight at 4°C. Immunoreactions were visualized using Alexa Fluor 488 or 555-conjugated secondary antibodies (1:200; Invitrogen). Mounting medium containing DAPI was then applied. Images were acquired using a fluorescence microscope (Nikon, Japan). For quantitative comparison of the expression of indicated molecules, the percentage of the positively double-stained area to the total traced area was determined in triplicate.

### Western blotting

Platelets were lysed with RIPA buffer (Cell Signaling) and then supplemented with a protease inhibitor cocktail (Sigma). Following SDS-PAGE, proteins were transferred to polyvinylidene difluoride membranes and probed with primary antibodies (from Cell Signaling) against phospho-p38, p38, phospho-Akt, Akt, and β-actin (Sigma). Immunoreactivity was detected with ECL (Pierce).

### Real-time RT-PCR

Total RNA was extracted from pooled carotid arteries using TRIzol Reagent (Invitrogen) and treated with DNase I to remove genomic DNA. Quantitative real-time reverse-transcriptase (RT)-PCR was performed with a Bio-Rad thermocycler and SYBR green kit (Bio-Rad) according to the recommendations of the manufacturer. The sequence-specific primers used for the reaction are presented in Table 1. The relative mRNA expression was normalized by GAPDH RNA levels. Data are expressed as the fold change compared with normal common carotid arteries.

**Table 1:** Primer sequences for quantitative RT-PCR.

Primer	Primer sequence	PCR product size
ICAM-1	F: 5'-CCTGCCTAAGGAAGACATGA-3' R: 5'-CCCAGACTCTCACAGCATCT-3'	[222 bp]
VCAM-1	F: 5‘-CCCAAACAGAGGCAGAGTGT-3' R: 5‘-CAGGATTTTGGGAGCTGGTA-3'	[150 bp]
TNF-α	F: 5’-CCCACTCTGACCCCTTTACT-3’ R: 5’-TTTGAGTCCTTGATGGTGGT-3’	[201bp]
IL-6	F: 5’-CTACCCCAATTTCCAATGCT-3’ R: 5’-ACCACAGTGAGGAATGTCCA-3’	[187bp]
GADPH	F: 5’-CTGGAGAAACCTGCCAAGTA-3’ R: 5’-TGTTGCTGTAGCCGTATTCA-3’	[223bp]

### Flow cytometry analysis of P-selectin, CD154, and CD147 expression on platelets

Washed platelets were pre-incubated with vehicle control, Akt1/2 inhibitor (Akti 1/2, 5μmol/L, sigma), or p38 MAP kinase inhibitor (SB203580, 10μmol/L, Cell Signaling) or left alone for 5 min at 37°C. Afterwards, platelets were stimulated with ADP (20 μmol/L) for an additional 15 min at 37°C. Samples were fixed in 1% buffered paraformaldehyde and then stained with antibodies against P-selectin-FITC (BD Bioscience), CD147-PE (eBioscience), CD154-PE (Pharmingen), or isotype control antibodies and analyzed by flow cytometry (FACSCanto II, BD Biosciences).

### Platelet-leukocyte interactions

Platelet-leukocyte aggregate formation was studied by flow cytometry. In vivo, blood was collected via a tail cut before (0 h), 2 h, and 4h after PLCA. In vitro, washed platelets were prepared from PRP, resuspended in HEPES buffer, and activated with ADP (20μmol/L) at 37°C. Leukocytes were isolated from the sediments obtained after the centrifugation and removal of PRP. After lysis of erythrocytes (Pharmlyse kit, BD Biosciences), leukocytes were washed twice with ice-cold Hanks HEPES buffer, added to resting or activated platelets, and incubated for 20 min at 37°C to generate platelet-leukocyte aggregates. Samples were stained with antibodies against CD45-Percp (BD Bioscience), Ly6G-APC (eBioscience), CD11b-PE (Pharmingen), CD41-FITC (Pharmingen), or isotype control antibodies and then analyzed by flow cytometry (FACSCanto II, BD Biosciences).

### Platelet-EC adherence assay

Mouse ECs, obtained from ATCC, were grown in DMEM supplemented with 10% FBS, 100 U/ml of penicillin, and 100 μg/ml of streptomycin. ECs were seeded onto 96-well plates (3×10^4^/well) and were quiescent in serum-free DMEM containing Insulin-Transferrin-Selenium (Cellgro), 0.1 mg/ml of BSA (Sigma), and antibiotics for 72 h. Resting or ADP activated platelets were incubated for 10 min (room temperature) with the fluorochrome carboxyfluorescein diacetate succinimidyl ester (CFSE, final concentration 90μM; Molecular Probes). The fluorescently labeled platelet solution was centrifuged, resuspended in PBS, and protected from light. The EC monolayer was activated with TNFα (10 ng/ml) for 5 h and then washed twice, after which labeled platelets (3.0 × 10^5^ cells/well) were added for 30 min. The ECs were washed twice to remove loosely adherent or unattached platelets and then fixed in methanol. The number of bound platelets was determined by direct counting cells in four randomly chosen microscope fields (20×) on a fluorescence microscope.

### Statistical analysis

Data are presented as mean ± SEM and determined using either two-tailed t-test analysis or one-way ANOVA followed by Fisher’s exact test analysis. P values less than 0.05 were considered statistically significant.

## Results

### 1. Platelet PI3Kγ contributes to activated platelet-induced intima-media thickening after partial ligation

To investigate whether circulating activated platelets contribute to vascular remodeling after partial ligation and whether platelet PI3Kγ serves an important function in this process, we injected activated WT or PI3Kγ^-/-^ platelets into C57BL/6 mice via tail veins. Saline was injected as control. To determine the ratio of injected platelets to endogenous platelets after transferring, we injected CFSE-labeled activated WT or PI3Kγ^-/-^ platelets (2 × 10^8^) into C57BL6J mice, and CFSE^+^ platelets were determined by flow cytometry. The percentage of CFSE^+^ platelets was 10.5±1.5% of the total platelet population in circulation 24 h after injection and decreased after 5 days (S1 Fig).

We observed a significant increase in intima-media thickening in WT platelet-infused mice compared with the control. By contrast, no significant increase was observed in PI3Kγ^-/-^ platelet-infused mice compared with the control (Fig 1A and 1C). These observations indicate that activated platelets aggravated vascular remodeling after partial ligation and that platelet PI3Kγ deficiency abrogated this effect. Although WT and PI3Kγ^-/-^ platelets showed similar responses to thrombin stimulation in vitro, as determined by cell surface markers of platelet activation, including P-selectin, CD147, and CD154, there was a marginal increase in intima-media thickening by the injection of thrombin-activated PI3Kγ^-/-^ platelets into mouse, compared with the vehicle control (P = 0.08) (S2 Fig). This finding suggests that more complex mechanisms are involved in the contribution of platelet-derived PI3Kγ to intima-media thickening in vivo.

### 2. Platelet PI3Kγ mediates activated platelet-induced leukocyte recruitment after partial ligation

Recruitment of leukocytes to the injured arterial wall has been shown to be an initial step that is critical to vascular inflammatory response and neointima formation [22]. In this study, we examined the effects of activated platelets on leukocyte recruitment and explored the function of platelet PI3Kγ.

**Fig 2:**
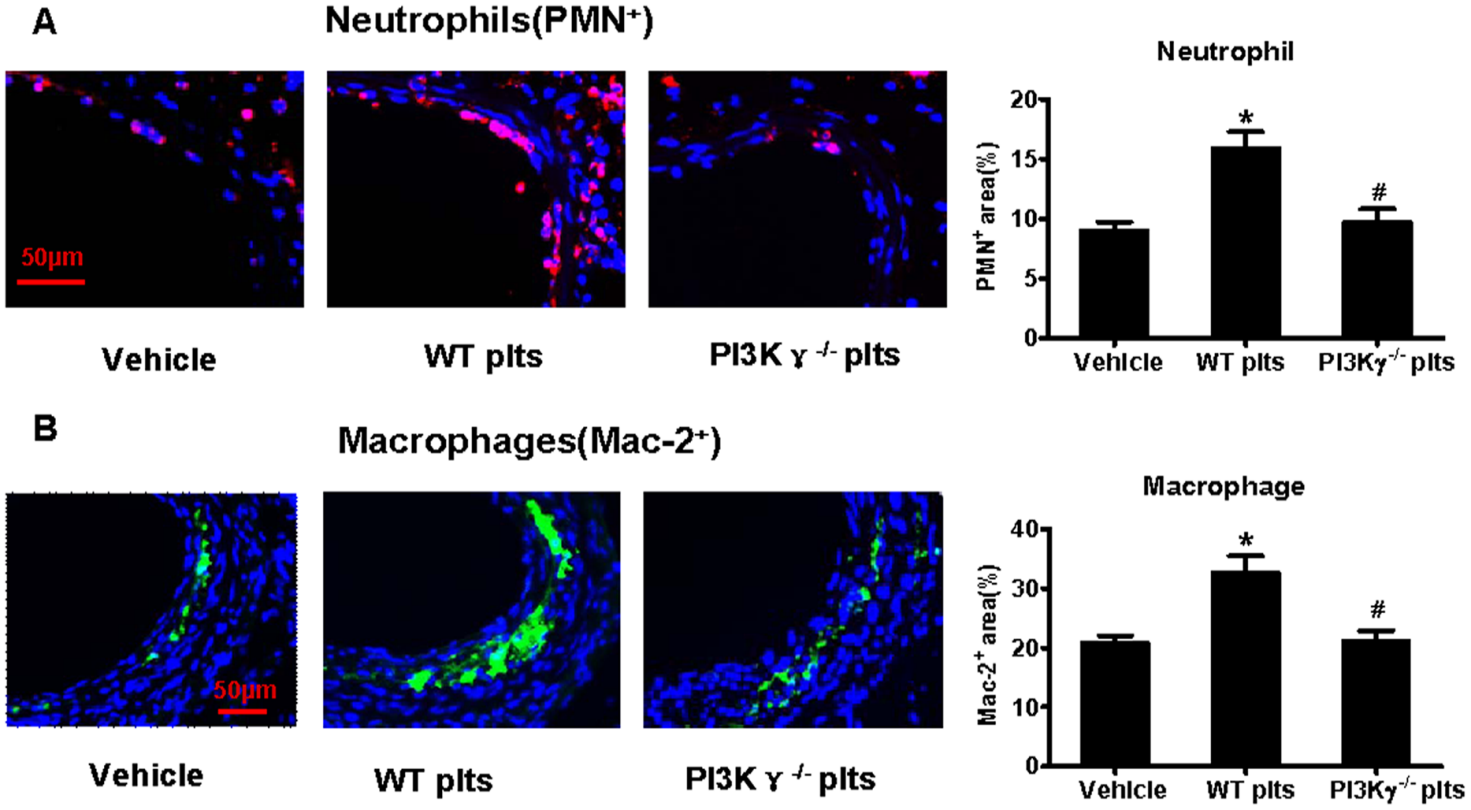
Platelet PI3Kγ mediates activated platelet-induced leukocyte recruitment after partial ligation. Representative images of PMN^+^ neutrophils (3 d) and Mac2^+^ monocyte/macrophages (21 d) immunofluorescence staining for left common carotid arteries from WT mice treated with PBS, WT platelets, or PI3Kγ^-/-^ platelets after partial ligation, as well as their quantitative analysis (A, B) (n = 5 per group). Scale bars: 50 μm. Data are expressed as mean ± SEM. * *P*<0.05 versus vehicle, # *P*<0.05 versus WT platelet-infused mouse.

In WT platelet-infused mice, neutrophil infiltration was significantly increased at 3d, whereas Mac-2^+^ macrophages were dramatically increased at 21 d. However, no significant change was observed in PI3Kγ^-/-^ platelet-infused mice compared with PBS-infused mice (Fig 2A and 2B). Our data showed that activated platelets increased leukocyte recruitment, whereas platelet PI3Kγ deficiency diminished this effect.

### 3. Platelet PI3Kγ contributes to activated platelet-induced overexpression of proinflammatory mediators in the carotid artery wall after partial ligation

To elucidate the molecular mechanisms underlying the effect of PI3Kγ-mediated activated platelets on leukocyte recruitment, we examined the expression of proinflammatory mediators in the flow-disturbed area.

**Fig 3:**
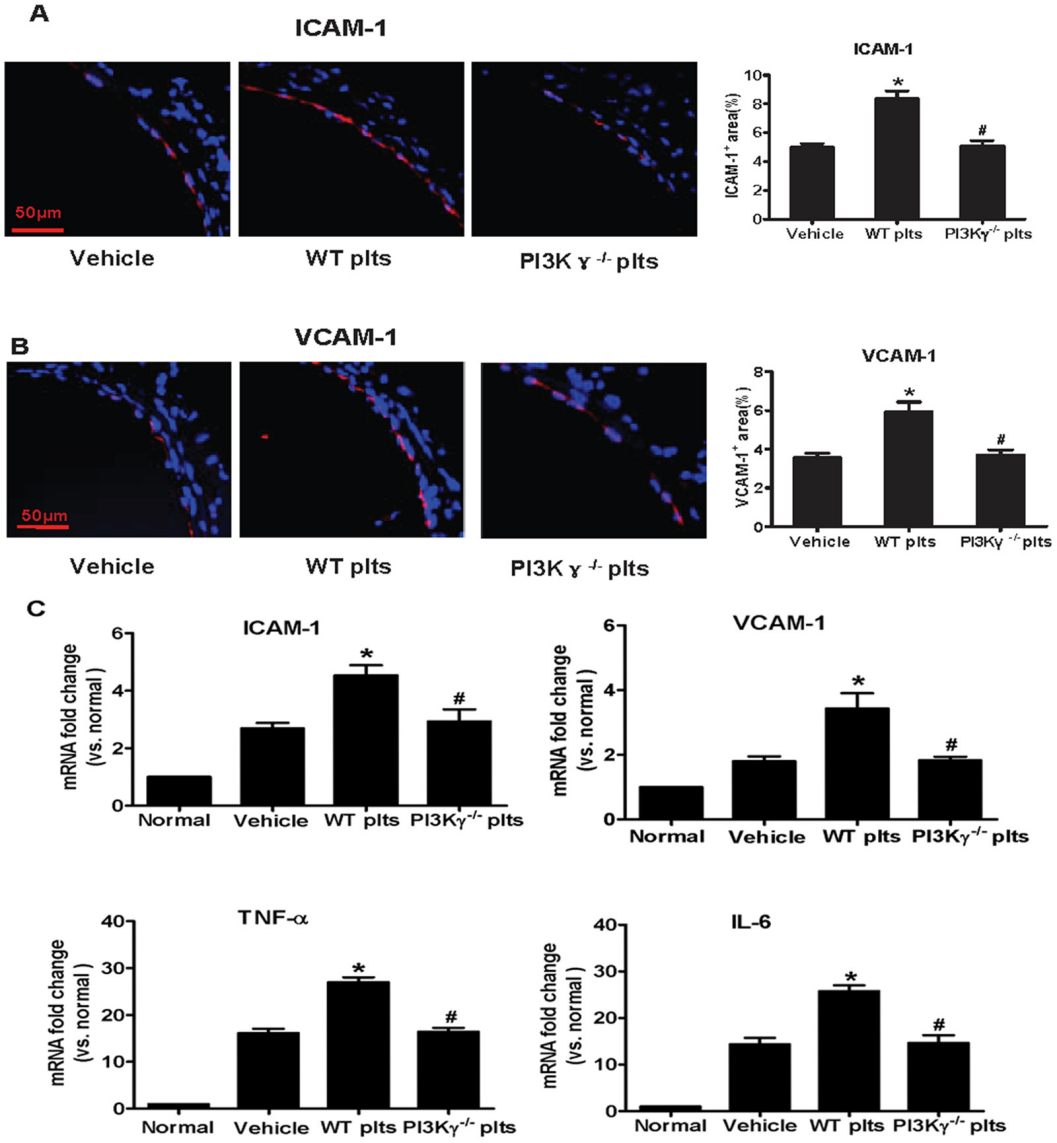
Platelet PI3Kγ contributes to activated platelet-induced overexpression of proinflammatory mediators in the carotid artery wall after partial ligation. (A, B) Representative images of ICAM-1 and VCAM-1 immunofluorescence staining for left common carotid arteries from WT mice treated with PBS, WT platelets, or PI3Kγ^-/-^ platelets after partial ligation at 3 d, as well as their quantitative analysis (n = 5 per group). Scale bars: 50 μm. (C) mRNA levels of VCAM-1, ICAM-1, TNF-α, and IL-6 were determined by quantitative RT-PCR in left common carotid arteries from WT mice treated with PBS, WT platelets, or PI3Kγ^-/-^ platelets after partial ligation at 3 d (n = 5 per group). mRNA levels are normalized to GAPDH. * *P*<0.05 versus vehicle group, # *P*<0.05 versus WT platelet-infused mouse.

Immunofluorescence staining demonstrated that the expression of adhesion molecules (ICAM-1 and VCAM-1) was markedly increased 3 d after injury in WT platelet-infused mice (Fig 3A and 3B), but the increase was markedly blocked in PI3Kγ^-/-^ platelet-infused mice (Fig 3A and 3B) compared with PBS infused mice. The regulation of ICAM-1 and VCAM-1 expression was confirmed by real-time RT-PCR analysis (Fig 3C). Moreover, real-time RT-PCR analysis demonstrated that the mRNA levels of proinflammatory cytokines tumor necrosis factor (TNF)-a and interleukin (IL)-6 were markedly increased in the carotid arteries of WT platelet-infused mice 3 d after partial ligation, but the levels were significantly lower in PBS or PI3Kγ^-/-^ platelet-infused mice (Fig 3C). These cytokines are known to stimulate the expression of various adhesion molecules and chemokines potently in vascular cells and leukocytes. Therefore, platelet PI3Kγ contributes to activated platelet-induced overexpression of proinflammatory mediators in response to disturbed flow.

Taken together, our data provide the first direct in vivo evidence that platelet PI3Kγ is critical role to the mediation of proinflammatory gene expression, leukocyte infiltration, and further vascular remodeling in response to blood flow disturbance.

### 4. Platelet PI3Kγ is required for the GPCR agonist ADP-induced platelet activation

**Fig 4:**
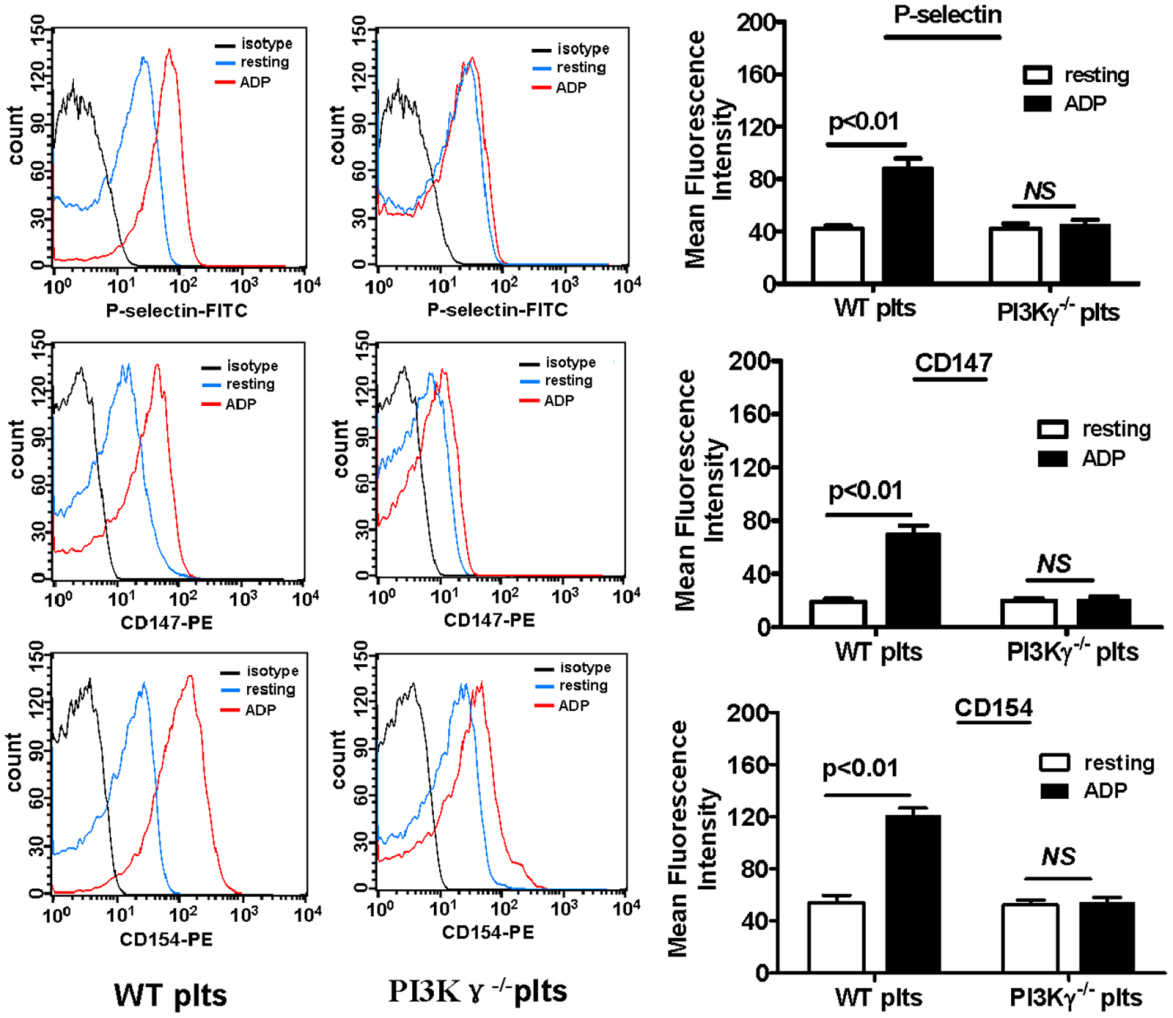
Platelet PI3Kγ is required for the GPCR agonist ADP-induced platelet activation. Representative flow cytometric analyses of P-selectin, CD147, and CD154 expressions on platelet surface, as well as the statistical data analyses from three separate experiments (n = 5 per group). Data are expressed as mean ± SEM.

To explore further the function of PI3Kγ in platelet function in vitro, we examined the activation of WT and PI3Kγ^-/-^ platelet response to ADP. We used flow cytometry to detect CD154, P-selectin, and CD147 expressions on the surface of WT and PI3Kγ-null platelets. Data showed that the expressions of CD154, P-selectin, and CD147 in WT platelets significantly increased after ADP stimulation, but no change was observed in PI3Kγ-null platelets (Fig 4). However, there was no significance between WT platelets and PI3Kγ^-/-^ platelets in response to thrombin stimulation in vitro (S2 Fig). This finding is consistent with a previous report [23].

### 5. Platelet PI3Kγ mediates ADP-induced platelet activation through Akt- and p38 MAPK-dependent mechanisms

**Fig 5:**
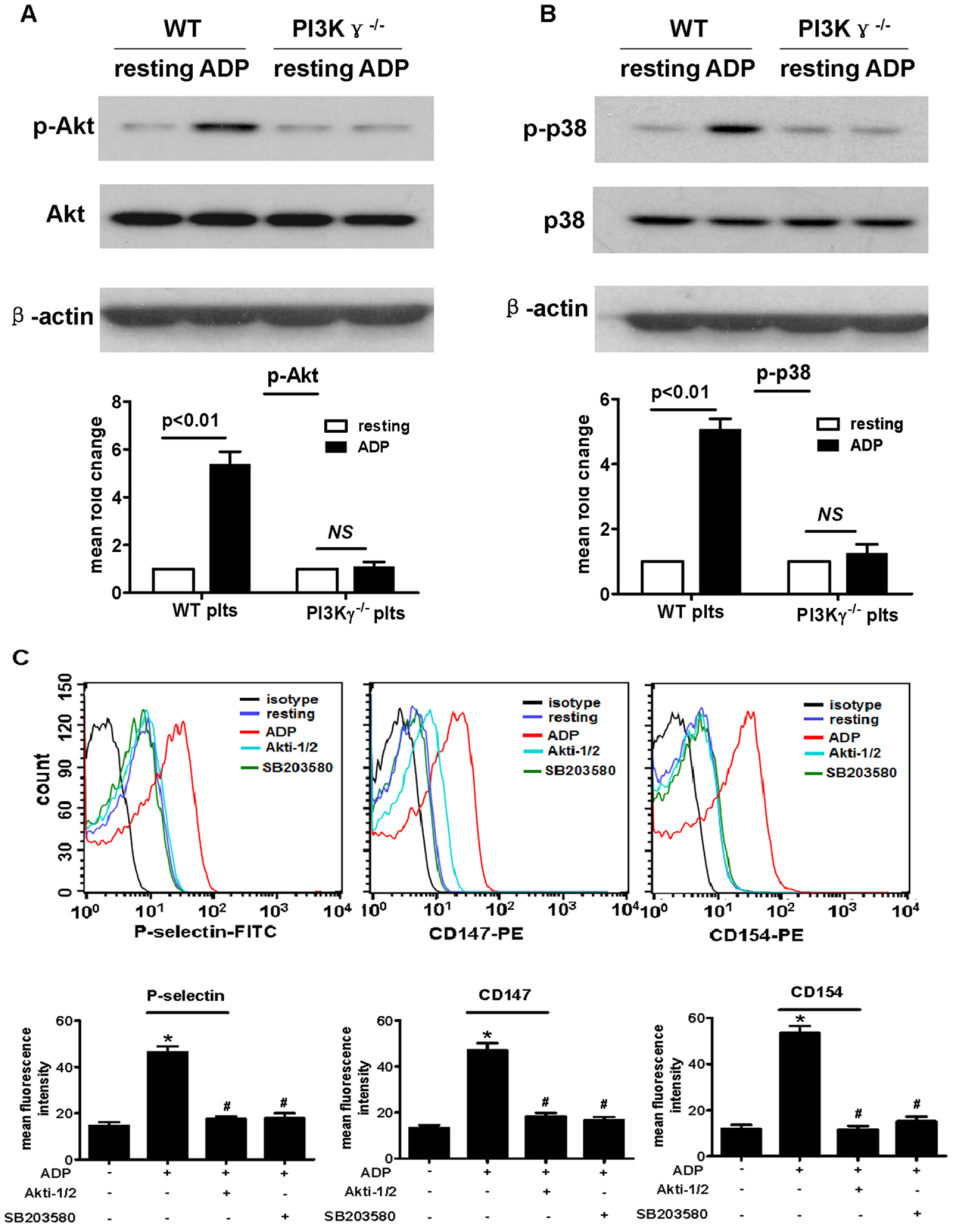
Platelet PI3Kγ mediates ADP-induced platelet activation through Akt- and p38 MAPK-dependent mechanisms. (A, B) Western blot analysis of phosphorylated p-p38 and p-Akt in platelets stimulated with ADP (20 μmol/L), with total p38 and Akt protein as internal controls. Average densitometric values normalized against those of internal control from three independent experiments are shown in the bar graph. Density from WT resting platelet sample was set as 1.0. Data are expressed as mean ± SEM. (C) Representative flow cytometric analyses of P-selectin, CD147, and CD154 expressions on WT platelet surface pretreated with Akti-1/2 (5 μmol/L) or SB203580 (10 μmol/L) for 5min, followed by stimulation with or without ADP (20 μmol/L) for 15 min, as well as the statistical data analysis from three separate experiments.

Akt and p38 MAPK have been established as important signaling intermediates in agonist-induced platelet activation, adhesion, and platelet ROS generation [24]. Therefore, we examined whether the Akt and p38 MAP kinase signaling pathways are involved in ADP- PI3Kγ-induced platelet activation. As shown in Fig 5A and 5B, Akt and p38 MAP kinase phosphorylations were significantly enhanced after stimulation with ADP in WT platelets. By contrast, no significant change was noted in PI3Kγ^-/-^ platelets. Akt1/2 inhibitor (Akti-1/2) and the p38 MAP kinase inhibitor SB203580 eliminated ADP-induced platelet CD154, P-selectin, and CD147 expression (Fig 5C). Taken together, these data suggest that platelet PI3Kγ mediates platelet activation through Akt- and p38 MAP kinase-dependent mechanisms.

### 6. Platelet PI3Kγ deficiency impairs platelet-leukocyte aggregation in vivo and in vitro

Activated platelets in circulation are known to bind leukocytes and form proinflammatory platelet-leukocytes aggregates (PLAs) [4, 5]. To investigate further the function of platelet PI3Kγ in the platelet-leukocyte interaction, we examined the platelet-neutrophil aggregates (PNAs) and platelet-monocyte aggregates (PMAs) in vivo and in vitro.

**Fig 6:**
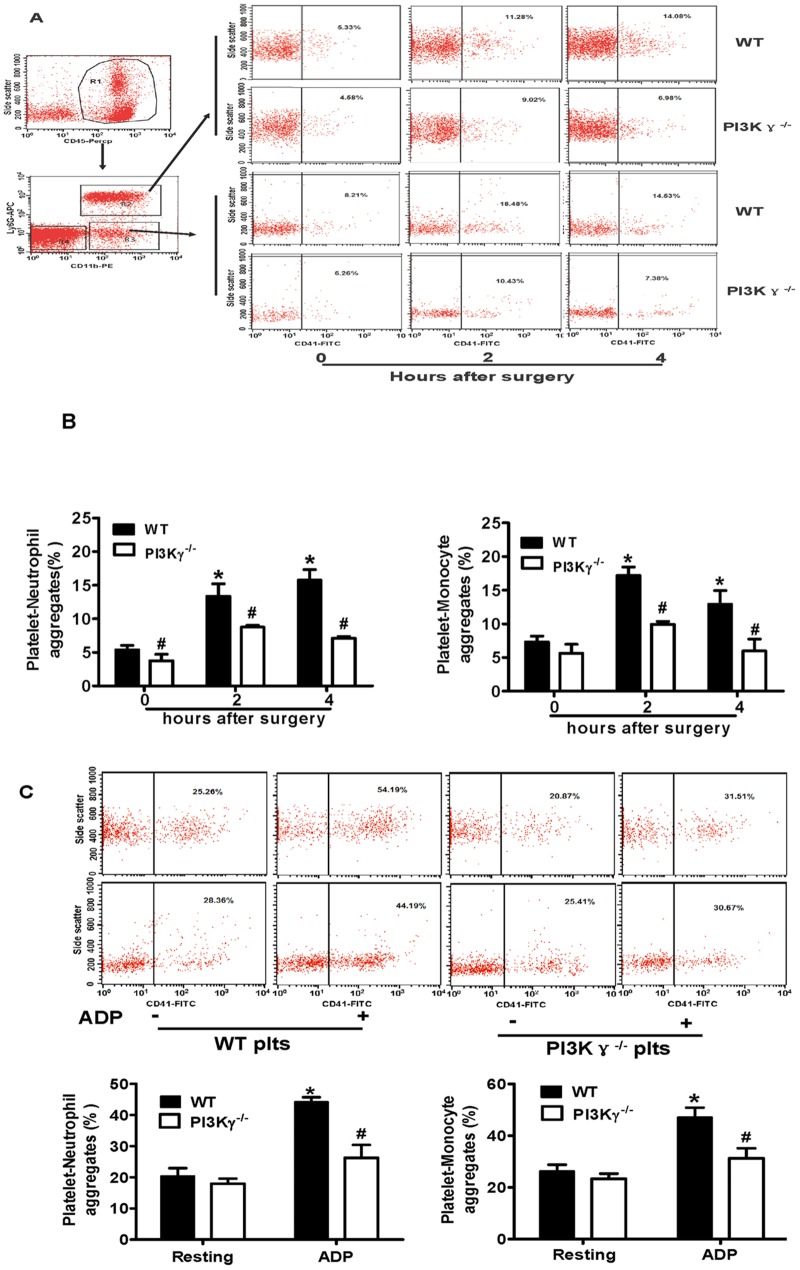
Platelet PI3Kγ deficiency impaired the platelet-leukocyte aggregates in vivo and in vitro. (A) Flow cytometry of leukocyte subpopulations, R1: CD45^+^ leukocyte, R2: neutrophils R3: monocytes. (A) Representative flow cytometric analyses of platelet-neutrophil aggregates (% total CD45+ Ly6G+ CD11b+ cells) and platelet-monocyte aggregates (% total CD45^+^ Ly6G^-^ CD11b^+^ cells) in WT or PI3Kγ^-/-^ mice before (0 h), 2 h, and 4 h after partial ligation in vivo, (B) as well as the statistical data analysis. (C) Representative flow cytometric analyses of platelet-neutrophil aggregates (% total CD45+ Ly6G+ CD11b+ cells) and platelet-monocyte aggregates (% total CD45^+^ Ly6G^-^ CD11b^+^ cells) in vitro, as well as the statistical analysis (n = 5 per group). Data represent mean ± SEM. * *P*<0.05 versus PI3Kγ^-/-^ group.

In vivo, we detected PNA and PMA formation in WT or PI3Kγ^-/-^ mice before (0 h), 2 h, and 4 h after PCLA. Flow cytometric analysis demonstrated that the PNA and PMA formations increased after PCLA both in WT and PI3Kγ^-/-^ mice, but the increase in PI3Kγ^-/-^ mice was significantly lower than that in WT mice (Fig 6A and 6B). In vitro, we analyzed the PLA formation between the ADP activated-WT or-PI3Kγ^-/-^ platelets and leukocytes isolated from C57BL/6 mice. Data showed that in WT platelets, the formation of PNAs and PMAs increased when activated with ADP. By contrast, in PI3Kγ^-/-^ platelets, no significant increase in PNA and PMA formation was observed after ADP stimulation (Fig 6C). These findings indicate that platelet PI3Kγ deficiency impaired the platelet-leukocyte interaction.

### 7. Platelet PI3Kγ deficiency impairs the platelet-EC interaction in vitro

**Fig 7:**
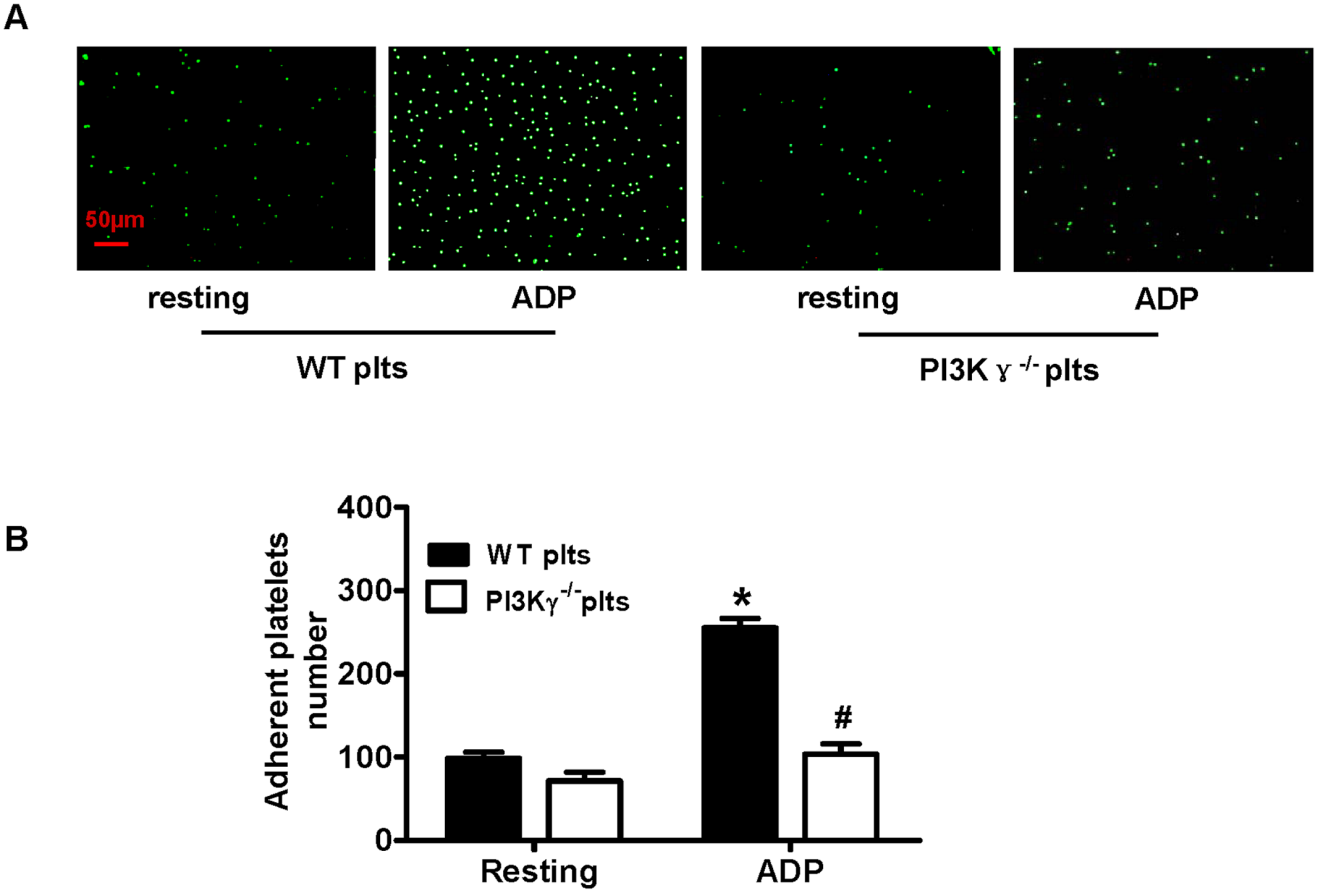
Platelet PI3Kγ deficiency impaired the platelet-EC interaction in vitro. (A) Representative fluorescent images of CFSE-labeled platelets adherent to ECs (n = 5 per group). Light microscope magnification is 10× (B) Statistical analysis of platelet adhesion measured as described in Materials and Methods. Data shown are mean ± SEM from three separate experiments. * *P*<0.05 versus resting platelets, # *P*<0.05 versus activated WT platelets.

To investigate the contribution of PI3Kγ to platelet-EC interaction in vitro, we analyzed the adhesion of WT or PI3Kγ^-/-^ platelets to activated ECs. We found more activated WT platelets, but not resting or PI3Kγ^-/-^ platelets, adherent to activate ECs (Fig 7A and 7B). This finding suggests that platelet PI3Kγ deficiency impaired the platelet-EC interaction.

## Discussion

This study presents several novel important findings as follows: First, activated platelets contribute to disturbed flow-induced vascular inflammatory response and CIMT, at least in part through platelet PI3Kγ. Second, platelet PI3Kγ is required for ADP-induced platelet activation through the Akt and p38 MAP kinase signaling pathways. Third, platelet PI3Kγ is required for ADP-induced proinflammatory PLA formation and platelet-EC interaction.

Platelets are best known as the cellular mediators of thrombosis. Emerging evidence indicates that platelets serve a major function in inflammation [25]. Platelets are found to be serve as both target and effector cells in inflammation response. A previous study found that activated platelets exacerbated atherosclerosis through the repeated injection of activated platelets into ApoE^-/-^ mice [5]. CIMT is a predictor of cardiovascular diseases, such as coronary artery disease and stroke. In this work, we demonstrated for the first time that repeated injection of activated platelets aggregated CIMT after blood flow disturbance.

CIMT is a well-recognized marker of atherosclerosis. Inflammation is involved in every stage of the progression of CIMT and atherosclerosis. Disturbed flow results in rapid endothelial dysfunction, leukocytes infiltration, and vascular vessel inflammation. Numerous proinflammatory mediators, including chemokines and adhesion molecules, can not only enhance vascular inflammation but also promote leukocyte recruitment. This study confirmed that activated platelets contribute to leukocyte recruitment and vascular inflammation following blood flow disturbance and serve to govern the disturbed flow-induced synthesis of a variety of proinflammatory mediators in the arterial wall, including chemokines (e.g., TNF-α and IL-6) and adhesion molecules (e.g., VCAM-1 and ICAM-1). Therefore, our results help explain the mechanism by which activated platelets contribute to vascular remodeling after blood flow severely reduced.

Recently studies suggest that PI3Kγ is a key regulator of inflammation and is implicated in inflammation-related diseases. In vitro and in vivo studies showed that PI3Kγ-deficient mice displayed impaired migration of neutrophils and macrophages toward chemoattractants [11–16]. After vascular injury, PI3Kγ-deficient mice and mice expressing catalytically inactive PI3Kγ exhibited reduced arterial occlusion and accumulation of monocytes and T cells around sites of vascular lesion [26]. PI3Kγ, which is known to be activated by GPCRs [27], is highly expressed in platelets and was found to be relevant to thrombosis. In murine blood, absence of PI3Kγ facilitated the formation of unstable thrombi, thereby leading to the dissociation of multiplatelet aggregates. PI3Kγ^-/-^ mice showed impaired platelet aggregation along with reduced thrombotic tendency. ADP, acting via GPCRs, is one of the most important mediators of platelet activation in vivo. ADP is locally secreted by activated platelets or released by damaged vascular wall cells and serves an important function in reinforcing the effects of other agonists. In the platelet aggregates formed after vascular injury, local ADP extracellular nucleotide concentrations may temporarily exceed 100 μmol/L [28]. ADP receptor inhibitors, such as thienopyridine, ticlopidine, and clopidogrel, are widely used in the treatment of cardiac and cerebrovascular diseases [29] relevant to atherosclerosis and atherothrombosis. Our study found that platelet PI3Kγ deficiency abrogated the activated platelet effects on leukocyte recruitment, vascular inflammation, and vascular remodeling after partial carotid ligation. These findings indicate that platelet PI3Kγ is important in vascular inflammatory response after flow disturbance. To investigate further the mechanism involved in this process, we explored the function of PI3Kγ in platelet function in vitro.

Activated platelets express numerous molecules, such as P-selectin, CD154, and CD147, which are well known to be involved in vascular injury, not only in activated platelet adhesion to dysfunctional ECs but also in the interaction with leukocytes, mediating leukocyte trafficking, and recruitment to the injured vessel [30,31]. We found that PI3Kγ^-/-^ platelets showed severely reduced P-selectin, CD154, and CD147 expression in response to ADP.

The Akt and p38 MAP kinase signaling pathways are important in platelet adhesion and activation. We found that these signaling pathways were involved in ADP-PI3Kγ-induced platelet activation. These results suggest that PI3Kγ mediates ADP-induced platelet activation through Akt and p38 MAPK-dependent pathways.

A large number of PLAs were observed in patients with acute myocardial infarction and unstable angina following angioplasty and other inflammation diseases [32, 33]. PLAs appear to be critical to the inflammation present in vascular diseases [34]. Activated platelets could indirectly support leukocyte recruitment via PLA formation [35]. We found that platelet PI3Kγ deficiency impaired the formation of PLAs in vivo and in vitro. CD154 and CD147 expressed by activated platelets were involved in PLA formation via their ligands in circulating leukocytes [36, 37]. The reduced expression of CD154 and CD147 on PI3Kγ-null platelets may cause the impaired formation of PLAs.

Moreover, we found that platelet PI3Kγ deficiency also impaired platelet adhesion to ECs in vitro. Increasing evidence suggests that activated platelets adherent to the inflamed endothelium may enhance leukocyte recruitment, activation, and transmigration, thereby enhancing the inflammatory processes in the vascular wall [38]. Studies found that P-selectin mediated platelet adhesion to ECs, with the involvement of CD147 and CD154 [26]. In the adjoining ECs, the platelet-secretory mediators alter the chemotactic, adhesive, and photolytic properties of the endothelium, further promoting the switch to an angiogenic, inflammatory, and thrombotic endothelial phenotype. The reduced expressions of P-selectin, CD154, and CD147 on PI3Kγ-deficient platelet response to ADP result in the impairment of PLAs, leukocyte recruitment, platelet-endothelial interaction, and vessel inflammation.

These findings explain why platelet PI3Kγ deficiency did not increase leukocyte recruitment and vascular remodeling under flow-disturbed conditions. The mechanisms by which platelets participate in inflammation response are very diverse. Platelet PI3Kγ blocking can inhibit platelet CD154, P-selectin, and CD147 expression and other molecules relevant to platelet function. The blocking is more widespread than the single inhibition of one surface molecule, such as CD154. Meanwhile, PI3Kγ mediates a large range of cellular responses, such as proliferation, survival, cytoskeletal remodeling, and membrane trafficking [39]. Platelet PI3Kγ specific inhibition may reduce major adverse effects. Therefore, platelet-specific PI3Kγ inhibition might be a potential target in the treatment of human cardiac and cerebrovascular diseases. Platelet-specific PI3Kγ-deficient mice are considered to evaluate further the function of PI3Kγ in platelet biology in vivo in inflammatory disease.

## Supporting Information

S1 Fig Platelet labeling in vitro and a platelet transfusion model in vivo. Representative flow cytometric analyses of CFSE+ platelets in the total circulation platelets 24 h and 5 day after injection.
(TIF)

S2 Fig Thrombin activated PI3Kγ^-/-^ platelets could not abolished the effect of activated platelets induced vascular remodeling after partial ligation. Platelets were activated by 0.2U/ml thrombin in vitro. (A) Representative Verhoeff’s elastic stained cross sections (level 3) of left common carotid arteries from WT mice treated with PBS (Vehicle), thrombin-activated WT platelets (WT plts), or thrombin-activated PI3Kγ^-/-^ platelets (PI3Kγ^-/-^ plts) (n = 5 per group) at 21 d after partial ligation. Intima+ media areas were measured at seven section levels (200μm intervals), and their mean values were determined. Data are expressed as mean ± SEM. * *P*<0.05 versus vehicle, # *P*<0.05 versus WT platelet-infused mouse. Light microscope magnification is 10×. (B-D) Representative flow cytometric analyses of P-selectin(CD62P), CD147, and CD154 expressions on platelet surface after thrombin activation, as well as the statistical data analyses from three separate experiments (n = 5 per group). Data are expressed as mean ± SEM.
(TIF)
