# Supplementary figures and images for: Platelet PI3Kγ Contributes to Carotid Intima-Media Thickening under Severely Reduced Flow Conditions
Source: PLoS One. 2015 Jun 8;10(6):e0129265. doi: 10.1371/journal.pone.0129265 (PMC4459692; doi:10.1371/journal.pone.0129265)

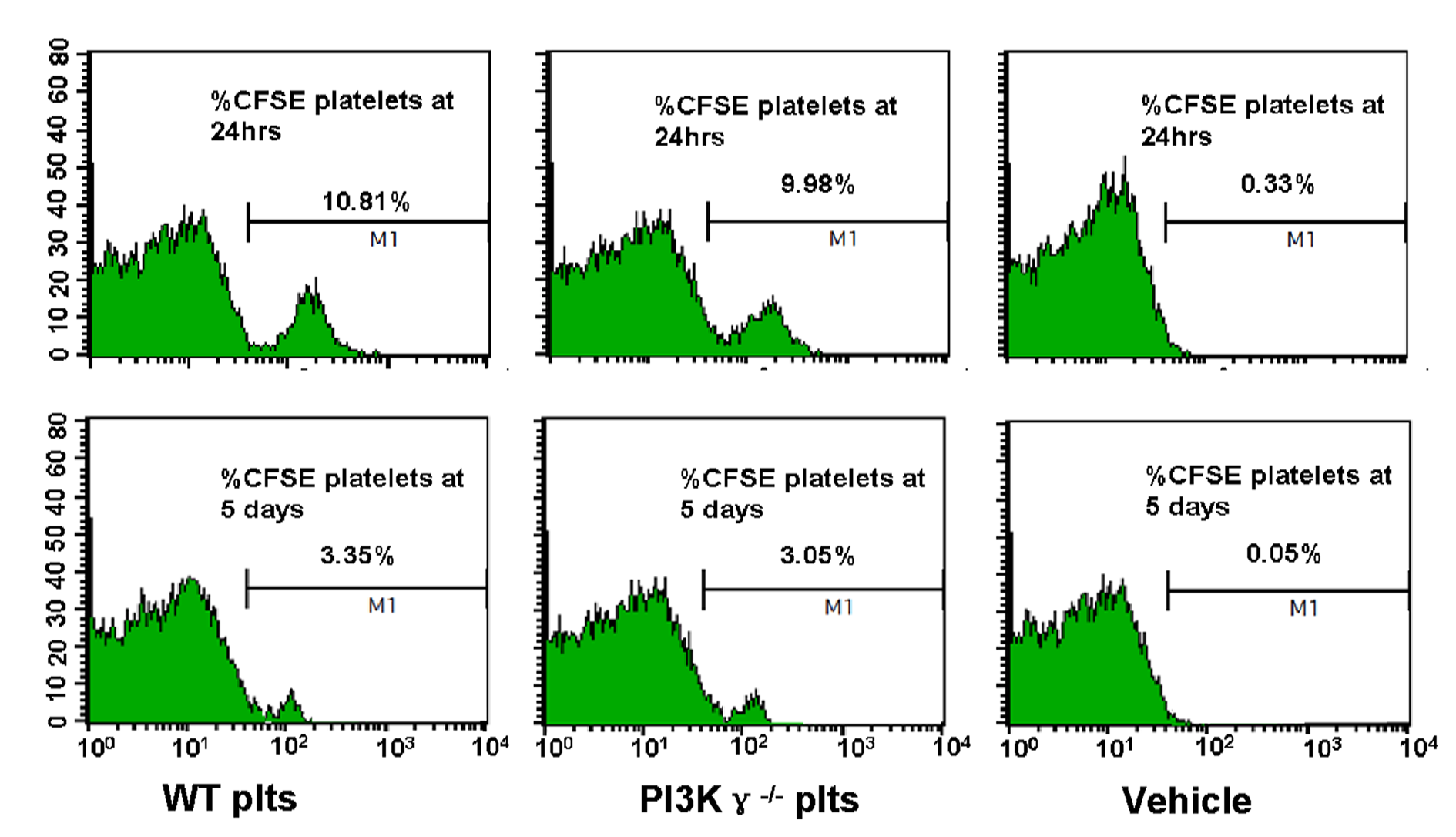

Supplement: S1 Fig — Representative flow cytometric analyses of CFSE+ platelets in the total circulation platelets 24 h and 5 day after injection. (TIF) [file pone.0129265.s001.tif]

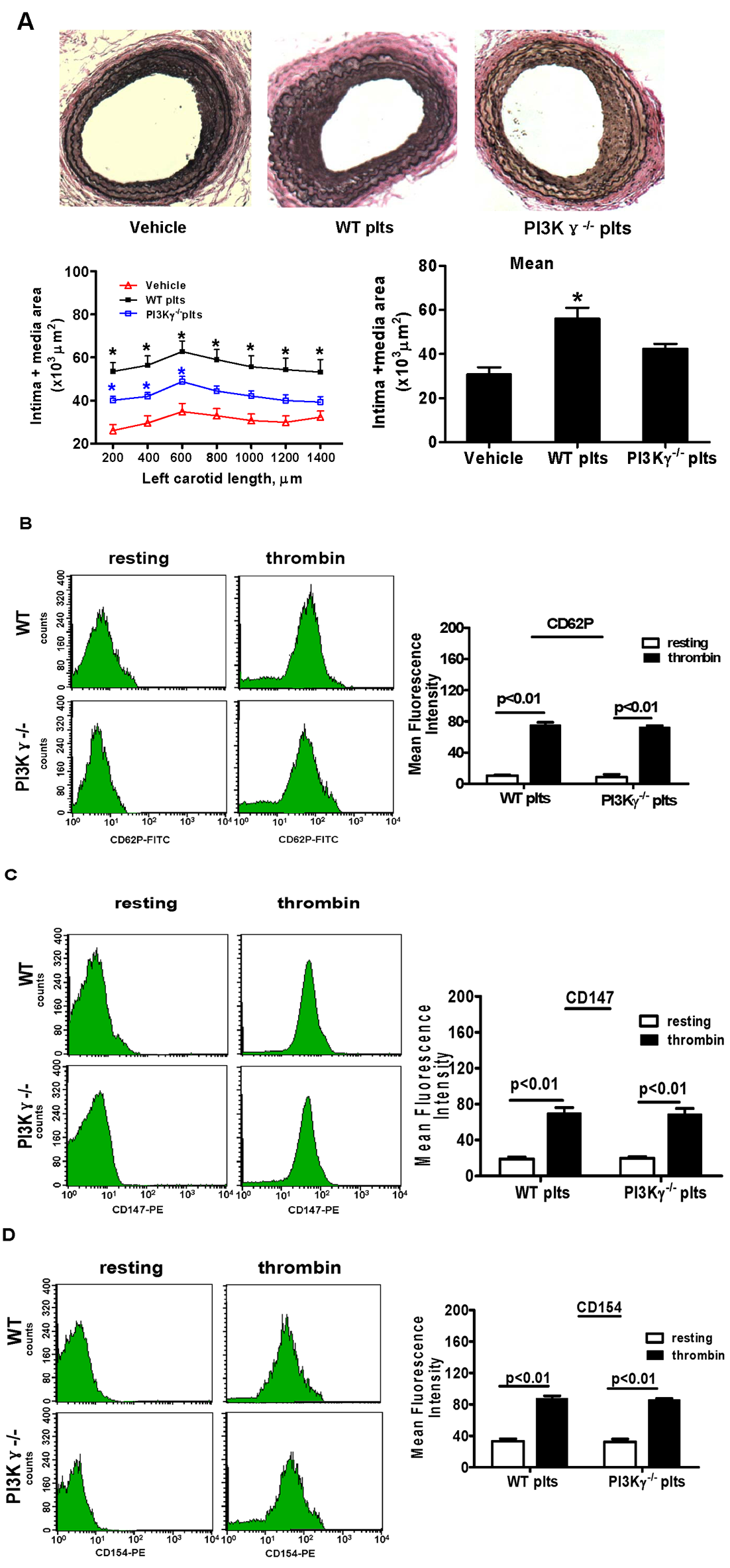

Supplement: S2 Fig — Platelets were activated by 0.2U/ml thrombin in vitro. (A) Representative Verhoeff’s elastic stained cross sections (level 3) of left common carotid arteries from WT mice treated with PBS (Vehicle), thrombin-activated WT platelets (WT plts), or thrombin-activated PI3Kγ-/- platelets (PI3Kγ-/- plts) (n = 5 per group) at 21 d after partial ligation. Intima+ media areas were measured at seven section levels (200μm intervals), and their mean values were determined. Data are expressed as mean ± SEM. * P<0.05 versus vehicle, # P<0.05 versus WT platelet-infused mouse. Light microscope magnification is 10×. (B-D) Representative flow cytometric analyses of P-selectin(CD62P), CD147, and CD154 expressions on platelet surface after thrombin activation, as well as the statistical data analyses from three separate experiments (n = 5 per group). Data are expressed as mean ± SEM. (TIF) [file pone.0129265.s002.tif]
